# Medical Progress

**Published:** 1894-05-05

**Authors:** 


### MEDICAL PROGRESS.

### RENAL AFFECTIONS.

*Diet.*—Perhaps the most prominent subject of discussion has been the diet and treatment of Bright's diseases. The reaction against a pure milk diet, especially in chronic disease, is shown in several recent utterances. Dr. Ralfe[1] read a carefully-guarded paper at the Medical Society, in which he showed—(1) that a pure milk diet in acute nephritis increased the quantity of urine, the amount of solids and urea, and decreased the albumen compared with what was excreted under other food; (2) that in chronic cases it produced a smaller increase of the urine, and even a decrease of the urea and other solids. If high tension was present it lessened the albumen, but if not, it had hardly any action in that respect. On the whole, he objected to its use except in acute cases and when he specially wished in chronic ones to reduce tension. Dr. Hale White agreed with this teaching, and said that albumen in most of the chronic patients in whom he had tested it had actually increased under such a diet, and though he did not consider albumen to be the really important factor, he feared the increased tendency to uræmia which was also noted in the same circumstances. His arguments for a mixed diet are given in the "Med. Chir. Trans.," Vol. LXXVI, The masterly paper contributed by Saundby[2] to the "Medical Annual" is a model of accurate thinking and clear epigrammatic expression. Albumen, he says, occurs in many other complaints, and its presence is in no way a measure of renal changes, even in Bright's disease. It is not evidence of a present inflammatory process, nor is its loss *per se* a serious drain to the system. Acute Bright's disease is curable, and the chronic forms may permit many years of fairly good health. The cardiovascular and retinal changes, the patient's general state, and the possibility of removing the cause should be the basis of our prognosis.

Milk, with farinaceous matters, is the staple food for the acute forms. For the chronic, "eat very sparingly of butcher's meat, avoid malt liquors, spirits, and strong wines," is the chief rule. When uræmia threatens cut off all butcher's meat, and for stout persons restrict the amount of food. Beef tea should be avoided in all cases. In acute exacerbations we may fall back on milk diet for short spells.

The value of a milk diet for a month after the febrile state in scarlatina, but modified wherever it is not otherwise well borne by the patient, has been referred to in the section on fevers. Lacorché and Talamon[3] condemn the indiscriminate use of milk alone in albuminuria; they point out that the adult requires seven pints daily to preserve the balance of tissue change, which is usually an impossible diet. Nor does a strict milk régime entirely remove albumen, but it is justifiable in acute nephritis and in acute exacerbations of chronic inflammatory disease, *i.e.*, when excessive diuresis is desired, and this régime should only be continued for a week or two. Dr. Dickinson[4] also, in an interesting paper, objects to a purely milk diet, even in acute nephritis, where he gives in addition bouillon and watery drinks *ad. lib.*, with farinaceous food. In chronic interstitial nephritis, instead of a purely milk diet, he insists on a moderate amount of meat and fish, with plenty of farinaceous food, milk, and large quantities of watery drinks. In every case alcohol must be reduced to a minimum. A warm climate, and very dry air are most important, and may give many years of health. For drugs he relies on the tartrates, citrates, digitalis, and saline purgatives, with occasional doses of calomel, but avoids other diuretics, and especially cantharides. In uræmia, frequent Turkish and lamp baths and pilocarpine. A lamp bath for weak patients may cover only the lower limbs. In syphilitic lardaceous disease iodides given for at least two years produce wonderful results.

*Drugs.*—The objection to *calomel* as a diuretic from its action on diseased kidneys, and the rapid salivation which then may occur, does not prevent it being a valuable remedy in renal dropsy, according to Dr. Dounine,[5] of Wassaw, who found it frequently efficacious in doses of three grains thrice daily for three or four days. He combined it with one-sixth grain of opium, and could discover no ill effects.

The long-continued administration of *strontium salts*, especially the lactate, would seem to produce an initial form of nephritis in healthy dogs (Dr. C. Falcone[6]). So, too, the temporary albuminuria, which is sometimes produced by radishes, asparagus, green tea, and severe exertion may be followed if the irritation is continued by the appearance of leucocytes and tubecasts. This shows according to Penzoldt[7] an abnormal permeability of the urinary filter. If they continue after rest in bed, and strict diet, we must conclude that nephritis is present, hence the need of repeated microscopic examination when a trace of albumen is found.

Walling[8] has tried an almost exclusive diet of lean meat in chronic nephritis. A little butter, toasted brown bread, and tea without sugar, was allowed. The strength improved, but if the diet was relaxed, the patient fell back and the albumen increased.

The action and value of *diuretin* are still discussed. E. Main[9] believes it has a direct and non-irritant effect on renal tissue, dependent on the soluble form of theobromine it contains. It has little effect on other organs. Dr. B. W. Richardson[10] found depression, somnolency, and a rise of temperature when it was taken in excess. A temporary diuresis was observed under it even at times when other drugs had failed.

The excretion and history of nature's diuretic, *urea*, is of constant interest. Kaufman,[11] as the result of thirty-eight analyses, denies that any difference can be detected between the amounts found in arterial and venous blood by the most delicate methods, and Kache[12] has argued against the malarial origin of yellow fever from the small amount of urea per ounce of urine in the former type of disease as compared with that in yellow fever.

Gordon Sharp[13] gives some cautions in using Spiegler's test for albumen. This test is formed of corrosive sublimate two parts, tartaric acid one, glycerine five, and distilled water fifty parts. The urine is acidulated with acetic acid, filtered if necessary, and dropped with a pipette on the surface of the test solution. A ring forms if albumen is present; but if chlorides are absent or much diminished, as in pneumonia or in the dilute urine of chronic nephritis, the test fails or develops slowly. If the chlorides are in excess it is possible that no precipitation may occur, and in some conditions tartaric acid itself may hinder the precipitation of albumen by mercury. In fact, the mercuric albumate is not always soluble in a fixed amount of sodium chloride.

Apart from the question of the action of *nitroglycerine* or sodium nitrite in Bright's disease, their value in controlling arterial tension depends largely upon the mode of administration. To obtain their good effects for a long period, Dr. D. D. Stewart[14] recommends frequent doses, with breaks of a few days, every three weeks to prevent the system becoming habituated. In late stages of the disease, when the nervous system is broken down by want of rest, small doses of *morphine* will often act as a diuretic, through its tonic effect on the nerves, when everything else fails. This is well brought out by W. Pepper,[15] who instanced a case where the urine increased from sixteen to 120 ounces under minute doses of morphine, combined with a little strychnia. The serious effects of uræmia on the nervous system occasionally show themselves in hemiplegic attacks, of which many instances are recorded. Its peculiarities are its variability, mobility, and the complete recovery which sometimes occurs.

In order to make a diagnosis from the more common causes of hemiplegia, previous symptoms of renal failure are important aids[15]. Thus vertigo, nausea, hyaline casts, or albumen found before the attack may be valuable. Albumen after the stroke may arise from cerebral hæmorrhage, and, of course, the hemiplegia from cerebral hæmorrhage produced in the course of interstitial nephritis is a very different matter. The first thing to be aimed at in treatment is the removal of the uræmic state.

The presence of a cloud of phosphates[16] usually attends some digestive disturbances, and sometimes a state of nervous worry. Such urine often contains merely an excess of alkaline carbonates, due, possibly, to the deficiency of hydrochloric acid in the stomach, or of bile. Other cases excrete an actual excess of phosphates, and some of these exhibit symptons of diabetes insipidus in a serious form, and become permanent. The pathology of this type is unknown. They are sometimes associated with nerve lesions, and no specific treatment has been found of value. The administration of phosphorus, Dr. Thorndike[17] remarks has shown no good results.

It is interesting to refer to Monti's account of diabetes insipidus. He considers that there are no abnormal constituents in the urine, and that it depends on a lesion of the sympathetic, caused by the virus of acute fevers, head and spinal injuries, severe stomach and intestinal disease, and fright. Those cases due to fevers or trauma recover soonest. Cold water should not be given. Valerian, sulphate of zinc, and ergot have been found sometimes beneficial.

The question of the origin of hyaline casts has been warmly debated of late, Ernst and Ribbert asserting that they are derived from blood serum coagulated and altered by its stay in the tubules, and Lubarsch asserting that they arise from degenerated epithelium. Now the Weigert[18] fibrin stain affects them, but other hyaline matters of non-fibrinous origin are equally stained by it. They occur often in chronic disease, and not in the spots where fibrin is found in acute nephritis, but Ribbert has actually produced these casts by cutting off the blood supply for a time. After the return of the blood, and before degeneration of the epithelium was marked, the tubules were found to be full of a serous exudate, which coagulated into ordinary hyaline casts when the kidney was exposed to heat.

Moreover, the presence of albumen has usually preceded the appearance of hyaline casts, if it does not co-exist with them, showing that exudation has occurred. Lubarsch,[19] however, insists that even here the casts are the result of degenerative changes, and not produced directly from inflammation.

The detection of bile pigments[20] in urine, even when present in minute proportion, can be made by adding a tenth of chloroform with a few drops of dilute HCL. After shaking, the sediment is run off and evaporated, when a marked colour shows the presence of bile. The nitric acid test may then be applied.

The diagnosis of disease by inspection of the urine without seeing the patient was a common folly in the time of Shakespeare,[21] over which he breaks many a jest. The great amount that can be learned, however, from a simple inspection of the urine, which is to-day frequently omitted altogether, is the subject of an interesting paper by Peyer, of Zurich. It is difficult, however, to summarise the many points which he refers to.

The abnormal forms of tubercular kidney[22] disease include one which is marked by renal colic without gravel bladder symptoms enlargement or tenderness of the kidney; and a hæmorrhagic one without pus or bacilli in the urine, or enlargement. A very rare form of sacculated kidney containing cholesterin in the calyx, was exhibited by Mr. H. Fenwick at the Pathological Society, and a conjoined, or horseshoe one, is described by Edington.[23] The left part of this specimen was divided into five lobes, and the right into four. The instances of general cystic degeneration of the kidneys which have been recorded recently are remarkable, as well as their compatibility with good health. Three were discovered in about six months at the Bristol General Hospital, and Collier[24] reports a fourth from Oxford. There was great enlargement, and the substance of the organs was almost entirely converted into closely aggregated cysts. Death in this case followed a hæmorrhage between the capsule and the kidney after the patient lifted some heavy weights. The bacteriology of cystic and surgical kidney has been examined by Krogius.[25] The B. coli communis is the most common invader, but in some cases without causing inflammation of the urinary tract. Cystitis when the B. coli was present alone was accompanied by acid urine, while that marked by Proteus vulgaris presented alkaline reactions. The gonococcus and staphylococcus pyogenes were also found.

Poitou-du-Plessy[26] considers that the careful administration of chloroform has no bad effects on diseased kidneys, or in uræmia convulsions. He prefers, however, a mixture of bromide of ethyl with chloroform.

The occurrence of unilateral retinitis albuminurica has been several times noticed. A similar instance is discussed by W. O. Moore,[27] where the left eye was markedly affected, the right, in which a cataract operation had been previously done, remaining normal, owing as he thinks to its diminished tension. He believes the changes to be degenerative and not inflammatory. Where great failure of sight has occurred in the albuminuria of pregnancy and is progressive, he advocates induction of labour, regarding the mother's life as in danger.

The action of *piperazine* on the excretion of uric acid when given in doses of 70 grains a day has been carefully examined by D. D. Stewart.[28] Great improvement of symptoms in uric acid calculi, gravel, and gouty arthritis had been previously noted with smaller doses, though the excretion of urea and uric acid and the acidity of the water were unaltered by them, but in some the amount of urine was markedly increased. A like effect on the urine was noticed in a case of chronic Bright's disease. In a case of gout when 70 grains daily were given no increase of uric acid, urea, or chloride could be made out, but only an augmentation of the water excreted, yet the joint pains diminished. On adding citrate of potash to the piperazine, the pains were rapidly cured and the swelling of the joints reduced. The potash alone had no effect. He suggests that the excess of uric acid was excreted as urea, the amount of which would not be thus perceptibly increased, or it may have passed out as allantoin. The clinical results at any rate were definite, but toxic symptoms appeared more than once with the large doses. Biesenthal also prevented the deposit of uric acid in the joints of pigeons, in which kidney lesions had been caused, by giving them piperazine.

Acute nephritis from sewer gas poisoning has been referred to previously. Vachell and Paterson[29] record four cases which were probably caused by the air from sewer ventilators in the street where all the patients lived. They occurred during dry weather, in which the smell from the sewer was particularly bad. Some showed ordinary symptoms, and one those of pyæmia in addition.

A report on the air of sewers has just been presented to the London County Council.[30] The bacteria are not pathogenic, and are derived from the outer air, not from sewage. On the whole it is much purer than was supposed, but may contain harmful chemical substances.

*Diabetes.*—The pancreatic origin of some cases of diabetes is illustrated by an instance where fatal diabetes developed in a patient of Hoppe-Seyler's.[31] The autopsy showed general arterio-sclerosis with fatty degeneration of the pancreas. The neighbouring vessels were calcified, the remaining glandular cells altered, and very few acini were to be found.

During most acute diseases the functions of the pancreas are diminished or lost. This has been confirmed by a long series of observations made by Vincent Harris[32] and Brown, and may be the case in diabetes not dependent on pancreatic disease. Hence one cause of the benefit produced by administering the juice of fresh pancreas. The administration of alkalies, especially by the subcutaneous or intravenous method, diuretics, and the inhalation of oxygen are again recommended by Harley[33] in coma. Bleeding freely is advocated by Huchard; subcutaneous saline injections and transfusion of blood are also suggested. He would prevent it by giving from four to ten drachms of bicarbonate of soda daily when there is any probability of its arising. Fatigue, diarrhœa, even an exclusive meat diet, and intestinal fermentation must be avoided, and cardiac tonics and purgatives should be given to guard against it.[34]

A pure carbohydrate diet containing no albumen was given to three normal and nine diabetic patients by H. Leo[35]. An increase in the urine and nitrogenous matter excreted in cases of moderate severity was found to result. Bullestini[36] reports cases of diabetes treated by large injections of pancreatic juice with success in reducing the excretion of sugar and improving the general condition of the patients.

Changes in the posterior columns of the spinal cord in diabetes, which were probably the result of the toxic condition of the blood, are detailed by R. T. Williamson.[37] The naked eye alterations, marked paleness, &c., were more evident than the microscopical. Only a very narrow rim of the myelin of many fibres in the posterior columns was stained black in Weigert preparations, the fibres being much swollen.

The treatment of the diabetic patient requires far more than mere attention to the sugar excreted. Indeed, Saundby[38] goes so far as to say "every one should be allowed as much carbohydrate as he is able to assimilate, estimating this by the amount of urine and sugar and the body weight," and "it is undesirable to keep diabetics who are losing flesh and strength on too strict a diet." He finds that milk (less than a pint) and two or three potatoes daily may often be added to their diet with little increase in the glycosuria and with benefit to the patient. He objects to gluten bread, but gives Clarke's biscuits, and a little toast in some cases. Iceland moss and lævulose are pleasant additions to the diet. Carbohydrates may actually actually remove acetone from the urine. Above all things constant watching and care of the general health is most important. Codeia phenacetin and the alkaline waters, with attention chiefly to the general health is urged by Vanderpoel,[39] who agrees in deprecating ordinary gluten bread. On a too rigid diet the nitrogenous structures may be broken down by the destructive metabolism more quickly than with a freer food supply. Bread from the soya bean and aleuronal have been found valuable substitutes for gluten flour. Aleuronal seems to be both cheap and palatable.

1 Lancet, Mar. 24, 1894. 2 Med. Annual, 1894. 3 Glasgow Med. J., Feb., 1894. 4 Lancet, Feb. 10, 1894. 5 Med. Week., Mar. 2, 1894. 6 Am. J. Med. Sc., Feb., 1894. 7 Practit., Feb., 1894. 8 Am. J. Med. Sc., Mar., 1894. 9 Am. J. Med. Sc., Mar., 1894. 10 Hospital, Mar. 3, 1894. 11 Med. Week., Feb. 9, 1894. 12 Lancet, Mar. 3, 1894. 13 Am. J. Med. Sc., Feb., 1894. 14 Inter. Clin., Vol. 3, 1893. 15 Med. Rec., Feb. 17, 1894. 16 Practit., Mar. 1894. 17 Deut. Med. Zeit., Dec. 18, 1893. 18 Centrall. f. Path. Bk., IV., 6, 11. 19 Fortsch. du Med., 1894. 20 Med. Week., Feb. 23, 1894. 21 Med. Week., Feb. 23, 1894. 22 Edin. Med. J., Feb., 1894. 23 Glasg. Med. J., Mar., 1894. 24 Bir. Med. Rev., Mar., 1894. 25 Am., J. Med. Sc., Mar., 1894. 26 Ther. Gaz., Jan., 1894. 27 Inter. Clin., Vol. 4, 1893. 28 Ther. Gaz,, Feb., 1894. 29 Lancet, Mar. 3, 1894. 30 Lancet, Mar. 24. 31 Med. Week., Mar. 9, 1894. 32 Bart.'s Hosp. Reports, 1894. 33 Ther. Gaz., Feb. 15, 1894. 34 Rev. Genl. de Clin., 46, 1893. 35 Inter. Med. Mag., Jan., 1894. 36 Prov. Med. J., Feb. 1, 1894. 37 B. M. J., Feb. 24, 1894. 38 Clin. J., Feb. 21, 1894. 39 N. Y. Med. J., Feb. 17, 1894.

